# Risk of bleeding associated with transbronchial biopsy using flexible bronchoscopy in patients with echocardiographic or chest CT evidence of pulmonary hypertension

**DOI:** 10.1186/s12890-022-02245-y

**Published:** 2022-11-28

**Authors:** Yuta Takashima, Naofumi Shinagawa, Daisuke Morinaga, Junichi Nakamura, Megumi Furuta, Tetsuaki Shoji, Hajime Asahina, Eiki Kikuchi, Junko Kikuchi, Jun Sakakibara-Konishi, Ichizo Tsujino, Satoshi Konno

**Affiliations:** 1 Department of Respiratory Medicine, Faculty of Medicine, Hokkaido University, North 15, West 7, Kita-Ku, Sapporo, 060-8638 Japan; 2 Division of Respiratory and Cardiovascular Innovative Research, Faculty of Medicine, Hokkaido University, Sapporo, Japan

**Keywords:** Bronchoscopy, Lung, EBUS, TBB, Echocardiography, Bleeding, Complication, Pulmonary hypertension

## Abstract

**Background:**

Endobronchial ultrasound (EBUS)-guided transbronchial biopsy (TBB) facilitates the diagnosis of various respiratory diseases. The safety of performing EBUS-guided TBB in patients with a finding of pulmonary hypertension (PH) is controversial. Little is known about the relationship between the risk of bleeding associated with EBUS-guided TBB in the presence of PH suspected on echocardiography or chest CT.

**Methods:**

To assess the risk of bleeding associated with EBUS-guided TBB in patients with presumed PH per echocardiography or chest CT, we retrospectively reviewed the medical records of 314 consecutive patients who underwent EBUS-guided TBB using a guide sheath (GS), as well as echocardiography and chest CT. Bleeding complication was defined as over one minute of suctioning; repeated wedging of the bronchoscope; instillation of cold saline, diluted vasoactive substances, or thrombin due to persistent bleeding. Findings of suspected PH were defined as peak tricuspid regurgitation velocity (TRV) > 2.8 m/s on echocardiography or pulmonary artery to aorta ratio (PA:A ratio) > 0.9 on chest CT.

**Results:**

In total, 35 (11.1%) patients developed bleeding, and all cases were managed safely. Furthermore, 17 (5.4%) and 59 (18.8%) patients were suspected to have PH based on echocardiography and chest CT, respectively. Among the patients suspected to have PH on echocardiography, five (5/17 = 29.4%) patients developed bleeding. Among the patients suspected to have PH on chest CT, 11 (11/59 = 18.6%) patients developed bleeding. Univariate analysis revealed that long diameter (≥ 30 mm) of the lesion, lesion location (the biopsy site was inner than the segmental bronchus), bronchoscopic diagnosis of malignancy, and additional biopsy were potential predictive factors for bleeding. The finding of suspected PH on echocardiography correlated significantly with bleeding (p = 0.03). On multivariate analysis, long diameter (≥ 30 mm) of the lesion (p = .021) and findings of suspected PH on echocardiography (p = .049) were significantly associated with bleeding.

**Conclusion:**

All cases of bleeding in the present study were managed safely. The risk of bleeding is moderately elevated when PH is suspected by echocardiography in patients undergoing EBUS-guided TBB using a GS.

**Supplementary Information:**

The online version contains supplementary material available at 10.1186/s12890-022-02245-y.

## Background

**Fig. 1:**
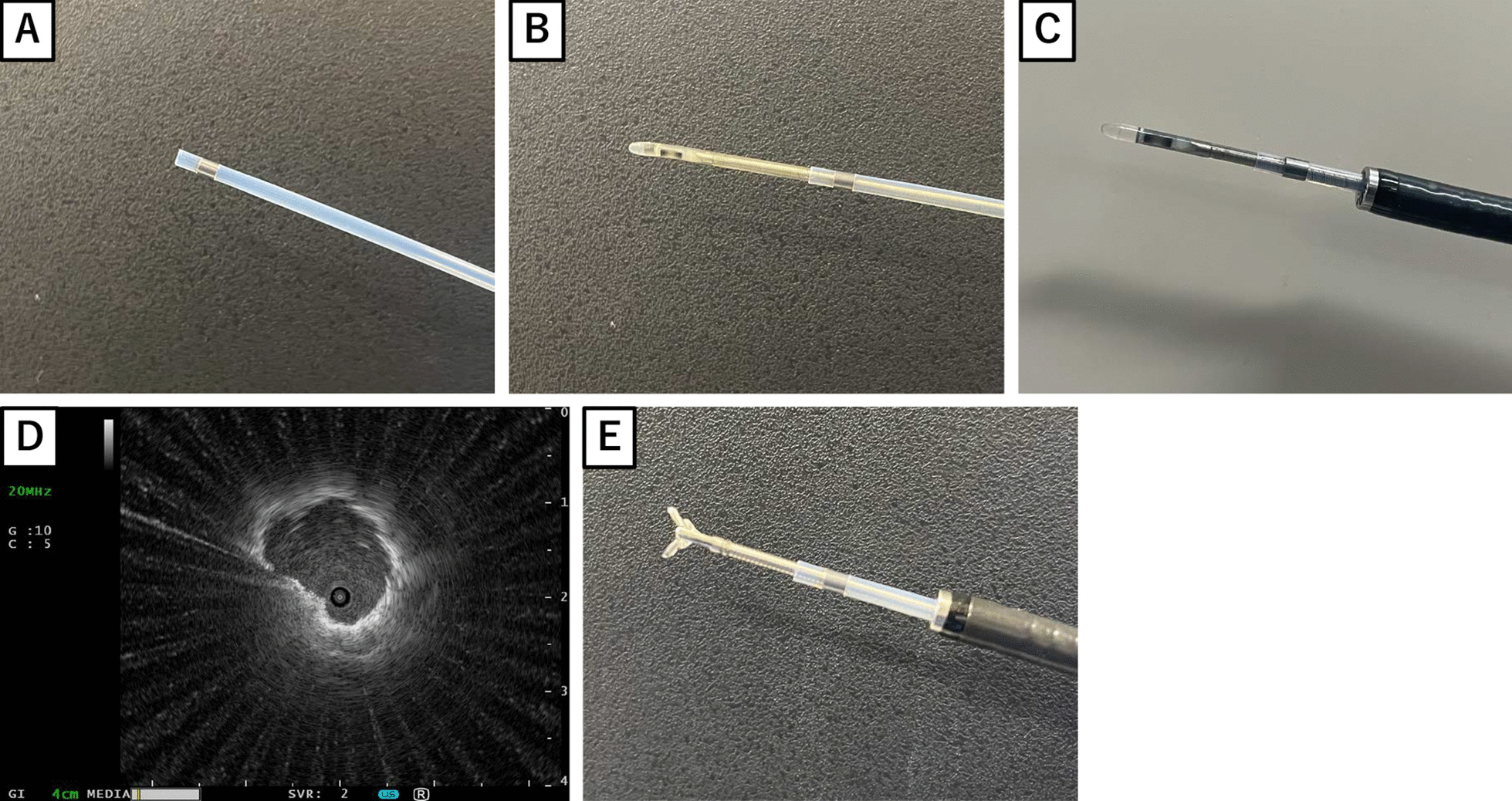
Endobronchial ultrasound (EBUS)-guide-sheath (GS)-guided transbronchial biopsy (TBB). **A** A picture of the GS. **B** The GS is attached to the ultrasound probe. **C** The ultrasound probe with GS is inserted through the working channel of the flexible bronchoscopy toward the target lesion. **D** The EBUS image is obtained when the ultrasound probe reaches the lesion. **E** The biopsy forceps are inserted into the GS after removing only the ultrasound probe

Flexible fiberoptic bronchoscopy is the main procedure used to diagnose and treat various respiratory diseases, including lung cancers, infections, and interstitial diseases. Endobronchial ultrasound (EBUS)-guided transbronchial biopsy (TBB) is an indispensable diagnostic procedure for peripheral lung lesions [1]. Several previous studies have also reported the usefulness of EBUS with a guide sheath (GS), in which biopsy forceps covered with a GS can be delivered to the lesions under EBUS guidance, and biopsy and brushing specimens can be sequentially obtained by keeping the GS in the lesion (Fig. 1) [2, 3]. Although a relatively safe procedure, EBUS-guided TBB using a GS (EBUS-GS-guided TBB) is rarely complicated by post-bronchoscopic adverse events such as bleeding, fever, pneumonia, and infection at the biopsy site. Bleeding associated with EBUS-GS-guided TBB is rarely reported; however, it constitutes one of the most serious procedural emergencies in daily practice. The reported risk factors for bleeding associated with bronchoscopic procedures include immunosuppression, mechanical ventilation, thrombocytopenia, lung transplant, anticoagulant therapy, antiplatelet therapy, and liver or kidney disease [4].

Pulmonary hypertension (PH) is an uncommon disease defined by elevated pulmonary arterial pressure (PAP). Prolonged PH leads to right ventricular dysfunction because of the sustained increase in right ventricular afterload. In the presence of added stresses, such as surgical interventions, patients with PH may fail to accommodate the changes in the right ventricular preload or afterload, at least in part due to alterations in the autonomic nervous system [5]. As a result, patients with PH are at a higher risk of intraoperative complications such as cardiac dysrhythmias and hemodynamic instability [6].

A previous study reported that a high PAP causes dilation of the submucosal bronchial veins and their plexuses, potentially increasing the risk of bleeding associated with TBB [7], and current recommendations suggest that TBB be performed with caution in patients with PH [8]. A few clinical studies examined whether PH increases the risk of bleeding with TBB, but the results were contradictory [9–13]. It is rare to perform TBB on a patient already diagnosed with PH in daily clinical practice; however, it is not uncommon to see PH be suspected based on echocardiography or chest computer tomography (CT) performed prior to TBB. Little is known about the relationship between the findings of suspected PH on such imaging modalities and the risk of bleeding complications associated with TBB.

Therefore, we conducted this retrospective study to assess the risk of bleeding associated with EBUS-GS-guided TBB in patients with suspected PH.

## Patients and methods

### Study patients

We retrospectively reviewed the medical records of 314 consecutive patients with EBUS-GS-guided TBB who underwent echocardiography between 6 months prior to and 1 month following bronchoscopy at the Hokkaido University Hospital, Japan, between January 2018 and June 2020. At our institution, all patients undergo a chest CT just before the EBUS-GS-guided TBB. We collected the following clinical information from all patients: age, sex, body mass index (BMI), smoking status, administration of antiplatelet agents or anticoagulant agents, CT scan features of target lesions (size and location), the requirement for additional biopsy using a larger size forceps or a flexible cryoprobe after GS removal, bronchoscopic diagnosis, which is the pathological diagnosis of specimens collected using a bronchoscope, and final diagnosis. Non-malignant outcomes, including the presence of atypical cells, fibrosis, and non-specific inflammation, were considered indeterminate results. These patients underwent further investigations, including surgical resection, to obtain a final diagnosis. The final diagnosis was considered as “undiagnosed” in the cases where EBUS-GS-guided TBB did not lead to a specific diagnosis and a follow-up chest CT was required. The medical ethics committee of the Hokkaido University School of Medicine approved the present study (Approval No. 021-0023).

### Bronchoscopic procedures

After providing local pharyngeal anesthesia using 2% lidocaine, all patients were sedated via IV administration of midazolam. In most patients, we administered IV fentanyl in combination with midazolam based on a previous report [14]. All bronchoscopies were performed with intrabronchial administration of 1% lidocaine. We used one of the following combinations of bronchoscopes and guide sheath kits (Olympus, Tokyo, Japan): BF-P260F or BF-P290 (working channel diameter 2.0 mm) with a K201 guide sheath kit, or BF-1T260 or BF-1TQ290 (working channel diameter 2.8 and 3.0 mm, respectively) with a K203 guide sheath kit. EBUS-GS-guided TBB was performed according to the standard method [3]. Before the procedure, we identified the target bronchus of the lesion and evaluated the bronchus sign on a thin-slice section of the chest CT [15]. Furthermore, in most cases, we used a virtual bronchoscopic navigation system, DirectPath® (Cybernet Systems, Tokyo, Japan). We used a reusable guiding device (CC-6DR-1; Olympus) in some cases to introduce a GS into the lesion via the target bronchus. After confirming the location of the lesion using EBUS and X-ray fluoroscopy, we procured at least six biopsy samples and performed bronchial brushing thrice. Per standard current practice, we attempted to acquire over 10 biopsy specimens for molecular and histochemical analyses. To obtain larger specimens, we conducted additional biopsy using a Radial Jaw 4 Standard Pulmonary Biopsy Forceps® 1.8 mm in some cases (Boston Scientific, Natick, MA, USA), which cannot be inserted into the GS due to its large cup size, or a Cryotherapy instrument, ERBE-CRYO2® (ERBE, Tubingen, Germany), with a flexible cryoprobe (1.9-mm diameter and 1,150-mm length) after GS removal. All biopsies were performed under X-ray fluoroscopy. After undergoing EBUS-GS-guided TBB, the patients routinely underwent chest X-ray the day after the bronchoscopy.

### Definition of bleeding complication

There is no consensus regarding the exact definition of bleeding associated with bronchoscopic biopsy, and it is often difficult to precisely calculate the blood loss during the procedure. In this study, bleeding was classified on a scale adapted from a previous report: [16] grade 1, suctioning of blood required for less than one minute; grade 2, suctioning required for more than one minute or repeat wedging of the bronchoscope for persistent bleeding or instillation of cold saline, diluted vasoactive substances, or thrombin; grade 3, selective intubation with endotracheal tube or balloon/bronchial blocker for less than 20 min or premature interruption of the procedure; grade 4, persistent selective intubation more than 20 min or new admission to the ICU or a need for packed red blood cell (RBC) transfusion, bronchial artery embolization, or resuscitation. In this study, we defined grade 2 or higher as a bleeding complication.

### Definition of findings of suspected PH on echocardiography and chest CT

**Fig. 2:**
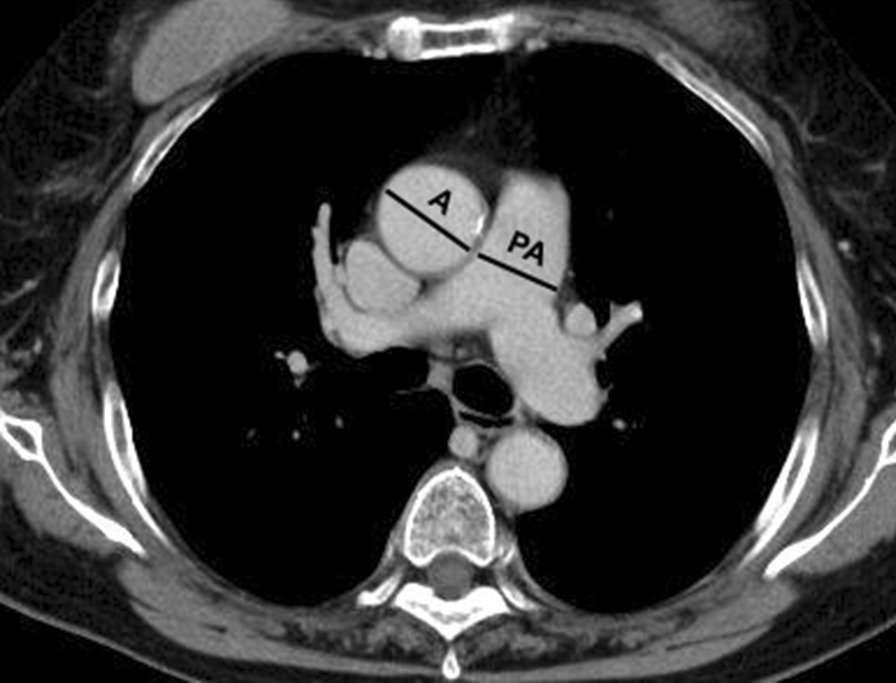
Axial chest CT image at the level of the left and right main pulmonary arteries. Measurements of the diameter of the main pulmonary artery (PA) and the diameter of the aorta (A) at the level of the bifurcation are used to calculate the PA:A ratio in accordance with a previous report [19]. If the diameter of A is not uniform, two measurements are taken 90 degrees apart, and the larger diameter is used

To identify patients with suspected PH, we assessed the results of echocardiography and chest CT. On echocardiography, we defined peak tricuspid regurgitation velocity (TRV) of > 2.8 m/s as a finding of suspected PH in accordance with previous reports [17, 18]. On chest CT, we measured the diameter of the main pulmonary artery at the level of its bifurcation and measured the maximum diameter of the ascending aorta using the same images, in accordance with previous literature (Fig. 2) [19]. We defined a ratio of the pulmonary artery to aorta ratio (PA:A ratio) of > 0.9 as a finding of suspected PH in accordance with previous reports [20, 21].

### Statistical analysis

The data are presented as median (range) or counts and percentages. Fisher’s exact test was performed to determine the association between the variables of interest and bleeding events. We used logistic regression models for multivariate analysis. Multivariate analysis was performed using factors that were significant in the univariate analysis. Two-sided *P* values < 0.05 were considered statically significant. All analyses were performed using the JMP software (JMP pro, version 15.2.0; SAS Institute Inc, Cary, NC, USA).

## Results

### Patient characteristics

The characteristics and clinical information of the patients at the time of EBUS-GS-guided TBB are summarized in Table 1. The median age of all patients was 71 years (range 38–88 years), and 192 of the 314 patients (61.1%) were men. In addition, 70 patients (22.3%) were never smokers. Although all cases had a drug holiday in accordance with the regulations of our department (Additional file 1: Table S1), the number of patients receiving antiplatelet agents and anticoagulant agents was 58 (18.5%) and 19 (6.1%), respectively. The median lesion size was 25 mm (range 7–92 mm). Lesions with positive bronchus sign localized inner than the segmental bronchus was seen in 37 patients (11.8%). The total number of malignant bronchoscopic and final diagnoses were 182 (58.0%) and 252 (80.3%), respectively.

**Table 1:** Characteristics of the patients (n = 314)

Characteristic	Value
*Age, years*	
Median (range)	71 (38–88)
< 75	222 (70.7)
≥ 75	92 (29.3)
*Sex*	
Male	192 (61.1)
Female	122 (38.9)
*BMI, kg/m*^*2*^	
Median (range)	23 (14.3–42.1)
< 25	215 (68.5)
≥ 25	99 (31.5)
*Smoking*	
Former	163 (51.9)
Current	81 (25.8)
Never	70 (22.3)
*Antiplatelet agent administration*^a^	
Yes	58 (18.5)
No	256 (81.5)
*Anticoagulant agent administration*^a^	
Yes	19 (6.1)
No	295 (93.9)
*Lesion size (long diameter), mm*	
Median (range)	25 (7–92)
< 30	187 (59.6)
≥ 30	127 (40.4)
*Lesion location*^b^	
Central	37 (11.8)
Peripheral	277 (88.2)
*Lesion location*	
Right upper lobe	100 (31.8)
Middle lobe	22 (7.0)
Lower lobe	65 (20.7)
Left upper lobe	68 (21.7)
Lower lobe	59 (18.8)
*Bronchoscopic diagnosis*	
Lung cancer and other malignancy	182 (58.0)
Benign	34 (10.8)
Unspecific	98 (31.2)
*Final diagnosis*	
Lung adenocarcinoma	159 (50.6)
Lung squamous cell carcinoma	42 (13.4)
Lung small cell carcinoma	12 (3.8)
Others (malignant)	39 (12.4)
Others (benign)	40 (12.7)
Undiagnosed^c^	22 (7.0)
*Additional biopsy after GS removal*^d^	
Yes	48 (15.3)
No	266 (84.7)

Values are No. (%) or as otherwise indicated

*BMI* Body mass index, *GS* guide sheath

^a^All cases set a drug holiday in accordance with the regulations of our department (Additional file 1: Table S1)

^b^Central lesion was defined as the lesion with bronchus sign positive localized inner than segmental bronchus

^c^Cases where EBUS-GS guided TBB did not lead to a specific diagnosis and follow-up chest CT was required

^d^Additional biopsy to obtain larger specimens using a Radial Jaw 4 Standard Pulmonary Biopsy Forceps® 1.8 mm (Boston Scientific, Natick, MA, USA) or a Cryotherapy instrument, ERBE-CRYO2® (ERBE, Tubingen, Germany), with a flexible cryoprobe (1.9 mm diameter and 1,150 mm in length) after GS removal

### Complications associated with EBUS-GS-guided TBB

The details of the complications associated with EBUS-GS-guided TBB are summarized in Table 2. In total, 35 patients developed bleeding, the cumulative incidence of which was 11.1% (35 of 314 EBUS-GS-guided TBB cases). Bleeding was the most common complication, and all bleedings were grade 2, requiring no interventional therapy or surgery. Among these patients, 28 were treated with thrombin spray through the working channel, and seven patients were treated with repeated wedging of the bronchoscope. Although no cases required prolonged hospitalization or postponement of the planned treatment, seven patients (2.2%) developed pneumothorax (none requiring drainage), and two patients (0.6%) developed an infection. In this study, the incidence of bleeding in patients receiving antiplatelet or anticoagulant agents prior to the bronchoscopic procedure was similar to that of the control group (data not shown). Furthermore, there were no adverse events associated with the discontinuation of the antithrombotic agents. No fatal complication was observed in this study.

**Table 2:** Complications associated with EBUS-GS-guided TBB

Complication	Value
Bleeding	35 (11.1)^a^
Pneumothorax	7 (2.2)^b^
Infection	2 (0.6)

Values are No. (%) or as otherwise indicated. *EBUS-GS-guided TBB* Endobronchial ultrasound with a guide sheath transbronchial biopsy

^a^Twenty-eight patients were treated with thrombin spray through the working channel and seven patients were treated with repeated wedging of the bronchoscope

^b^No case required drainage

### Findings of suspected PH on echocardiography or chest CT

The results of echocardiography and chest CT are summarized in Table 3. In echocardiography, the median peak TRV was 2.35 m/s (range 1.00–4.27 m/s). In chest CT, the median PA:A ratio was 0.79 (range 0.50–1.21). Among the 314 patients, 17 (5.4%) had a finding of suspected PH on echocardiography, while 59 (18.8%) had findings of suspected PH on chest CT (Table 3). Among the patients with findings of suspected PH on echocardiography, three patients underwent right heart catheterization prior to EBUS-GS-guided TBB. All three patients had a mean pulmonary arterial pressure (MPAP) of < 25 mmHg, which was not diagnostic of PH.

**Table 3:** The results of peak tricuspid regurgitation velocity (TRV) in echocardiography and pulmonary artery to aorta ratio (PA:A ratio) in chest CT

	Total (n = 314)	Non-bleeding (n = 279)	Bleeding (n = 35)
*Peak TRV, m/s*			
Median (range)	2.35 (1.00–4.27)		
Non-suspicious group (≤ 2.8)	297 (94.6)	267 (89.9)	30 (10.1)
Suspicious group (> 2.8)	17 (5.4)	12 (70.6)	5 (29.4)
*PA:A ratio*			
Median (range)	0.79 (0.50–1.21)		
Non-suspicious group (≤ 0.9)	255 (81.2)	231 (90.6)	24 (9.4)
Suspicious group (> 0.9)	59 (18.8)	48 (81.4)	11 (18.6)

Values are No. (%) or as otherwise indicated

A female patient with a peak TRV of 2.45 m/s and a PA:A ratio of 1.07 had already been diagnosed with chronic thromboembolic pulmonary hypertension (CTEPH) and had undergone pulmonary thromboendarterectomy (PEA). This patient did not develop bleeding. Among the patients with findings of suspected PH on echocardiography, five patients developed bleeding, an incidence of 29.4% (five out of 17 cases). Among the patients with a finding of suspected PH on chest CT, 11 patients developed bleeding, an incidence of 18.6% (11 out of 59 cases). Furthermore, among the 17 patients with findings of suspected PH on echocardiography, seven also had a finding of suspected PH on chest CT; four of these patients developed bleeding, an incidence of 57.1% (four out of seven cases).

### Risk factors for bleeding associated with EBUS-GS-guided TBB

To clarify the risk factors for bleeding associated with EBUS-GS-guided TBB, we investigated the clinical factors potentially related to bleeding. The relationship between various clinical factors and bleeding is presented in Table 4. Univariate regression analysis revealed that among these factors, a long diameter (≥ 30 mm) of the lesion, lesion location (biopsy site was inner than segmental bronchus), bronchoscopic diagnosis of malignancy, and additional biopsy were potential predictive factors. A finding of suspected PH on echocardiography correlated significantly with bleeding (p = 0.03). A finding of suspected PH on chest CT was more frequently observed in patients with bleeding compared with those without bleeding, although the difference was not statistically significant (p = 0.063). In multivariate analysis, a long diameter (≥ 30 mm) of the lesion (OR, 2.78; 95% CI, 1.17–6.62; p = 0.021) and a finding of suspected PH on echocardiography (OR, 3.44; 95% CI, 1.01–11.74; p = 0.049) were significantly associated with the development of bleeding.

**Table 4:** The relationship between clinical factors and bleeding complication

	Univariate analysis		Multivariate analysis	
	OR (95% CI)	P Value	OR (95% CI)	P Value
Age (≥ 75 years)	1.72 (0.83–3.55)	.168		
Sex (male)	0.56 (0.28–1.14)	.140		
BMI (≥ 25 kg/ m^2^)	1.74 (0.85–3.56)	.176		
Smoking (current or former smoker)	0.96 (0.42–2.23)	1.000		
Antiplatelet agent administration^a^ (yes)	0.90 (0.36–2.29)	1.000		
Anticoagulant agent administration^a^ (yes)	0.93 (0.21–4.22)	1.000		
Lesion size (≥ 30 mm)	4.34 (2.00–9.40)	< .001	2.78 (1.17–6.62)	.021
Lesion location^b^ (central)	5.30 (2.36–11.91)	< .001	2.11 (0.81–5.49)	.125
Lesion location (upper or middle lobe)	1.49 (0.70–3.15)	.361		
Bronchoscopic diagnosis (malignancy)	2.27 (1.03–5.04)	.045	1.30 (0.54–3.14)	.565
Final diagnosis (malignancy)	1.57 (0.59–4.24)	.502		
Additional biopsy after GS removal^c^ (yes)	3.52 (1.61–7.69)	.002	2.27 (0.96–5.41)	.063
TRV (> 2.8 m/s)	3.71 (1.22–11.25)	.030	3.44 (1.01–11.74)	.049
PA:A ratio (> 0.9)	2.21 (1.01–4.80)	.063		

*BMI* Body mass index, *GS* guide sheath, *TRV* tricuspid regurgitation velocity, *PA:A ratio* pulmonary artery to aorta ratio

^a^All cases set a drug holiday in accordance with the regulations of our department (Additional file 1: Table S1)

^b^The central lesion was defined as the lesion with bronchus sign positive localized inner than segmental bronchus

^c^Additional biopsy to obtain larger specimens using a Radial Jaw 4 Standard Pulmonary Biopsy Forceps® 1.8 mm (Boston Scientific, Natick, MA, USA) or a Cryotherapy instrument, ERBE-CRYO2® (ERBE, Tubingen, Germany), with a flexible cryoprobe (1.9 mm diameter and 1,150 mm in length) after GS removal

## Discussion

To our knowledge, this is the first retrospective study to show the risk of bleeding associated with EBUS-GS-guided TBB in patients with suspicion of PH by echocardiography. Some previous studies have examined whether a finding of suspected PH is a risk factor for bleeding. Consistent with our study, Lashari et al. reported that the incidence of bleeding was higher in the PH group, with an MPAP of ≥ 25 mmHg on right heart catheterization or a systolic pulmonary artery pressure (SPAP) of > 40 mmHg on echocardiography, which failed to reach statistical significance [13]. In contrast, Fuentes et al. reported that the incidence of bleeding in patients with findings of suspected PH on echocardiography, with SPAP of ≥ 36 mmHg, was equal to that of the control group [11]. With reference to Bernoulli's equation, which is used to calculate the tricuspid regurgitation pressure gradient (TRPG), our cut-off value is almost equivalent to theirs. The inconsistency between the results of our study and that of the study by Fuentes may be due to the differences in the anesthetic agent used, as well as the bronchoscopic procedures. In their study, the bronchoscopic procedures included TBB and endobronchial ultrasound-guided transbronchial needle aspiration (EBUS-TBNA), half of which were performed under general anesthesia provided by anesthesiologists. In contrast, in our study, all bronchoscopic procedures were EBUS-GS-guided TBB and performed under conscious sedation provided by bronchoscopists. While bronchoscopic procedures are generally performed under conscious sedation in Japan, general anesthesia is considered more suitable when strict control of vitals is required. Ishiwata et al. reported that the incidence of bleeding in patients with findings of suspected PH on echocardiography, with SPAP of > 40 mmHg, was similar to that in the control group [12]. However, approximately 40% of the procedures in their study consisted of bronchoalveolar lavage (BAL), which is associated with a lower frequency of postprocedural bleeds. Therefore, our study provides valuable information regarding the bleeding risks in patients with suspected PH, particularly considering that all patients in our study underwent EBUS-GS-guided TBB.

The cumulative incidence of bleeding in this study (11.1%) may be higher than that in previous reports [22–24]. This discrepancy may be due to the varying definitions of bleeding and different contexts in each study. Since any bleeding has the potential to be severe, we decided to define bleeding broadly to include even mild cases and consider whether we should pay close attention to bleeding when considering the adaptation of TBB for patients with suspected PH. In this study, every case of bleeding was managed safely, and none progressed to become life-threatening. The results of this study suggest that there is no absolute contraindication for TBB in patients with suspected PH; however, it is imperative that we pay close attention when considering the adaptation of TBB for such patients. Previous studies have suggested that the use of GS may reduce bleeding complications as the GS can compress the biopsy site after the biopsy [2, 25]. When performing TBB without a GS in patients with suspected PH, clinicians should be particularly careful about the risk of bleeding.

PH is defined as an increase in MPAP of ≥ 25 mmHg. Direct pressure measurement with right heart catheterization is the reference method and the “gold standard” for diagnosis of PH. However, a noninvasive assessment of SPAP by cardiac doppler echography is feasible and represents a keystone examination in suspected PH according to the current guidelines [18]. Since few patients undergo right heart catheterization before bronchoscopy in daily practice, the present study targeting patients with suspected PH on echocardiography and chest CT is valuable and highly applicable to real life.

Our study has several limitations. First, selection bias may be at play given that this is a single-center retrospective study. Although a multicenter prospective study is needed to confirm our results, this study is the first to provide real clinical data about the bleeding risks associated with EBUS-GS-guided TBB in patients with suspected PH and our findings could be valuable in the clinical setting. Second, only three patients with suspected PH on echocardiography underwent right heart catheterization prior to EBUS-GS-guided TBB in this study. We could not examine the association between the findings of suspected PH on echocardiography or chest CT and right heart catheterization. Third, echocardiography was not performed on the same day as TBB; however, echocardiograms were obtained within two weeks before the bronchoscopy in 67.2% of patients. Furthermore, the total number of major complications was low; therefore, using a stricter cut-off for the interval between echocardiography and bronchoscopy is not expected to have changed the outcome. Fourth, due to the significant amount of missing blood sampling data obtained at the time of TBB, we could not examine the association between bleeding complications and the blood sampling data, such as hemoglobin level, platelet count, and prothrombin time-international normalized ratio (PT-INR). Fifth, given the retrospective nature of this study, we are not able to comment on the technique and adequacy of sampling of each pulmonary biopsy.

## Conclusion

All cases of bleeding in the present study were managed safely. The risk of bleeding is moderately elevated when PH is suspected by echocardiography in patients undergoing EBUS-GS-guided TBB.

## Supplementary Information

**Additional file 1: Table S1.** Withdrawal periods of antiplatelet and anticoagulant agents for transbronchial biopsy in our department.

## Data Availability

The data used and/or analyzed during the present study are available from the corresponding author on reasonable request.

## Abbreviations

EBUS: Endobronchial ultrasound
TBB: Transbronchial biopsy
GS: Guide sheath
PH: Pulmonary hypertension
PAP: Pulmonary arterial pressure
CT: Computed tomography
BMI: Body mass index
RBC: Red blood cell
TRV: Tricuspid regurgitation velocity
MPAP: Mean pulmonary arterial pressure
CTEPH: Chronic thromboembolic pulmonary hypertension
PEA: Pulmonary thromboendarterectomy
SPAP: Systolic pulmonary artery pressure
TRPG: Tricuspid regurgitation pressure gradient
TBNA: Transbronchial needle aspiration
BAL: Bronchoalveolar lavage
PT-INR: Prothrombin time-international normalized ratio
