# Supplementary material for: Risk of bleeding associated with transbronchial biopsy using flexible bronchoscopy in patients with echocardiographic or chest CT evidence of pulmonary hypertension
Source: BMC Pulm Med. 2022 Nov 28;22:449. doi: 10.1186/s12890-022-02245-y (PMC9706867; doi:10.1186/s12890-022-02245-y)
Supplement: Supplementary file 1 — Additional file 1: Table S1. Withdrawal periods of antiplatelet and anticoagulant agents for transbronchial biopsy in our department. [file 12890_2022_2245_MOESM1_ESM.docx]

Table S1. Withdrawal periods of antiplatelet and anticoagulant agents for transbronchial biopsy in our department

| Drug | Drug withdrawal period |
| --- | --- |
| Antiplatelet agents |  |
| Clopidogrel sulfate | 7 days |
| Ticlopidine hydrochloride | 7 days |
| Prasugrel hydrochloride | 7 days |
| Acetylsalicylic acid | 7 days |
| Ethyl icosapentate | 7 days |
| Omega-3-acid ethyl esters | 7 days |
| Cilostazol | 3 days |
| Beraprost sodium | 2 days |
| Sarpogrelate hydrochloride | 2 days |
| Limaprost alfadex | 2 days |
| Dipyridamole | 1 day |
| Anticoagulant agents |  |
| Warfarin potassium | 3 days |
| Dabigatran etexilate | 1 day |
| Rivaroxaban | 1 day |
| Edoxaban tosylate hydrate | 1 day |
| Apixaban | 1 day |
